# Association between LXR-*α* and ABCA1 Gene Polymorphisms and the Risk of Diabetic Kidney Disease in Patients with Type 2 Diabetes Mellitus in a Chinese Han Population

**DOI:** 10.1155/2020/8721536

**Published:** 2020-12-22

**Authors:** Peng Liu, Liang Ma, Hailing Zhao, Zhengri Shen, Xuefeng Zhou, Meihua Yan, Tingting Zhao, Haojun Zhang, Xinping Qiu, Ping Li

**Affiliations:** ^1^Shunyi Hospital, Beijing Traditional Chinese Medicine Hospital, Beijing, China; ^2^Clinical Laboratory, China-Japan Friendship Hospital, Beijing, China; ^3^Beijing Key Lab for Immune-Mediated Inflammatory Diseases, Institute of Clinical Medical Science, China-Japan Friendship Hospital, Beijing, China

## Abstract

We designed a case-control study and selected *LXR-α* rs7120118 C>T and *ABCA1* rs2230806 A>G polymorphisms to determine the correlation between these polymorphisms and diabetic kidney disease (DKD) susceptibility in a Chinese Han population. Three hundred DKD patients and 346 type 2 diabetes mellitus (DM) patients without kidney disease were recruited. Our results showed that rs7120118 was associated with DKD (genotype, *P* = .027; allele, *P* < .011). rs7120118 was associated with a higher risk of DKD under a dominant model adjustment by age and sex (*P* = .015) and an additive model (*P* = .040); rs2230806 was associated with a higher risk of DKD under an recessive model (*P* < .03); the combined effect of rs7120118 CC+rs2230806 GG genotype showed an association of DKD adjustment for age and sex (*P* = .009). In subgroup analysis of patients without hypercholesterolemia, the rs2230806 genotype frequencies were different between the two groups (*P* = .042). rs2230806 was associated with increased risk of DKD under a recessive model adjustment for age and sex (*P* = .013) and an additive model (*P* = .031). Our results suggest that *LXR-α* rs7120118 is significantly associated with a higher risk of DKD, and *ABCA1* rs2230806 is significantly associated with a higher risk of DKD without hypercholesterolemia in Chinese Han individuals.

## 1. Introduction

Diabetic kidney disease (DKD), a devastating complication of diabetes mellitus (DM), is the most common cause of end-stage renal disease (ESRD) and renal failure in the world [1]. According to the International Diabetes Federation, in 2017, there were an estimated 425 million cases of adult DM worldwide, with more than 30% of these cases reported in China [2]. Genetic factors are directly related to the initiation and progression of DKD, including aggregation in families and variable incidence rates between different races [3, 4]. Hence, it is imperative to identify the potentially susceptible genetic *loci* of DKD in the prediction and prevention of DKD [5].

Lipid metabolism disorders are an important factor that leads to DKD progression [6]. As metabolite-sensing receptors, liver X receptors (LXRs) exist in two isoforms: LXR-*α* (encoded by the NR1H3 gene) and LXR-*β* (encoded by the NR1H2 gene) [7]. Physiologic processes that are affected by LXRs include inflammation, metabolism and homeostasis of lipids, and cholesterol homeostasis [8]. LXR*α* could upregulate the expression of hepatic lipogenic enzymes and increase blood TG levels, and LXR-*α*-deficient mice display markedly prevented hepatic fatty acid synthesis and triglycerides [9]. Multiple common single-nucleotide polymorphisms (SNPs) in LXR-*α* are associated with a higher risk of coronary heart disease and hemodialysis [10, 11]. Patients carrying the allele T (i.e., CT or TT) at rs7120118 have low serum lipid levels, while those with the C allele have high serum lipid levels in coronary heart disease and hemodialysis [10, 11]. Furthermore, it has been shown that rs7120118 is associated with the expression of LXR-*α* [12].

LXR-*α*, which function as the core determinants of cellular cholesterol homeostasis, induce expression of the cholesterol efflux transporter ATP-binding cassette subfamily A member 1 (ABCA1) to promote cellular cholesterol efflux [13]. As the most prominent member of the ATP-binding cassette family, ABCA1 is highly important for mediating cholesterol efflux from cells [14]. Pedigo et al. found that patients with DKD had decreased expression of ABCA1 impaired cholesterol efflux in macrophages and podocytes [15]. Ganda et al. showed that increased ABCA1-mediated cholesterol efflux evoked tubular cholesterol accumulation in patients with DKD [16]. ABCA1 is involved in high-density lipoprotein cholesterol (HDL-C) biogenesis, and changes in ABCA1 structure and/or protein expression could alter metabolic disturbances [17]. The loci of the ABCA1 gene rs2230806 is located in the major extracellular rings of the ABCA1 protein, which have an important role in APO-I and cholesterol efflux [18]. rs2230806 is also the most widely studied common missense polymorphism, and studies in Asians reported that *ABCA1* SNP (rs2230806, also known as R219K or G1051A) is associated with type 2 DM and coronary heart disease [19, 20].

However, there are limited studies regarding the susceptibility of LXR-*α* and ABCA1 polymorphisms in Chinese DKD populations. Therefore, because of the heterogeneity for gene polymorphisms in Han Chinese, we tested the hypothesis that there is an association of *LXR-α* rs7120118 (C>T) and *ABCA1* rs2230806 (A>G) with DKD in this population.

## 2. Materials and Methods

### 2.1. Patients

This was a case-control study that consisted of 646 volunteers. Three hundred participants had a history of type 2 DM and DKD. They were compared with 346 participants diagnosed with type 2 DM for at least 7 years and had no history of DKD. All participants were recruited from the China-Japan Friendship Hospital (Beijing, China). The study took place from February 2015 to October 2018. Diagnosis of type 2 diabetes mellitus was based on the World Health Organization 1999 criteria [21]. Criteria for DKD were defined by the National Kidney Foundation Kidney Disease Outcomes Quality Initiative (NKF K-DOQI) guidelines [22]. This study was approved by the institutional ethics committee of the China-Japan Friendship Hospital, and informed written consent was obtained from all participants.

### 2.2. Data Collection, DNA Isolation, and Genotyping

Clinical characteristics of age, sex, body mass index (BMI), duration of diabetes, blood pressure, serum creatinine (Scr), total cholesterol (TC), triglyceride (TG), low-density lipoprotein cholesterol (LDL-C), and HDL-C of each participant were obtained from the medical records.

Genomic DNA was isolated from whole blood using a QIAamp DNA Blood Mini Kit (Qiagen; Hilden, Germany) following standard procedures and quantified using a UV-visible spectrophotometer (NanoDrop 1000; Thermo Scientific, Waltham, MA, USA). Patient samples were genotyped using the TaqMan SNP genotyping assay (Applied Biosystems; Waltham, MA, USA) and the ABI PRISM 7500 Sequence Detection System (Applied Biosystems) as described previously [23].

A total volume of 25 *μ*L reaction mixture was used, containing 12.5 *μ*L 2× Taq Master Mix (Takara; Shiga, Japan), 50 ng DNA, 3 pmol of each probe (Applied Biosystems) to perform polymerase chain reaction (PCR) amplification of LXR and ABCA1. The PCR conditions consisted of 40 cycles of 92°C for 15 seconds, and 60°C for 60 seconds after incubation at 95°C for 10 minutes. All primers used to detect SNPs were synthesized by Applied Biosystems. To confirm the accuracy of genotyping, randomly selected PCR products were verified by DNA sequencing performed by TsingKe Biological Technology (Beijing, China), and the results were confirmed by TaqMan genotyping. Sequences of primers used for PCR were rs7120118, 5′-TGTGCTGCCTGGATGTATTG-3′ (forward) and 5′-CTCTGAGGGTC TGCTGATGC-3′ (reverse), and rs2230806, 5′-GTGTCCTGTCATTGTGCCTTGT-3′ (forward) and 5′-CTCCCAGCCAGCCGTACTTT-3′ (reverse).

### 2.3. Statistical Analyses

As non-Gaussian distributed data, clinical data were presented as median and interquartile range and underwent chi-square testing to detect differences in patient clinical characteristics. Hardy-Weinberg equilibrium analysis of both SNPs and the genotype and allelic frequencies of SNPs were compared using the chi-square test. Association between each SNP and susceptibility to DKD were estimated by multivariate logistic regression analysis of three genetic models (additive, dominant, and recessive), correcting for age and sex. Next, multivariate logistic regression analysis was also used to evaluate the combined effect of both *LXR-α* rs7120118 and *ABCA1* rs2230806 polymorphism on DKD. An example to define these genetic models is the rs7120118 SNP, where T is the minor allele and C is the major allele. An additive genetic model was assumed, coding CC, TC, and TT as the number of copies of the minor allele (0, 1, or 2). In the dominant model, TT and TC were coded as 1, and CC was coded as 0. In the recessive model, TT was coded as 1, and TC and CC were coded as 0.

## 3. Results

### 3.1. Baseline Characteristics

In total, 646 participants were included in this study. The study consisted of two groups: DKD patients (*n* = 300; males 184, females 116) and DM patients (*n* = 346; males 199, females 145) (Table 1). All clinical characteristics including BMI, history of hypertension, blood pressure, Scr, TC, TG, and LDL-C were found to be elevated in DKD patients as compared with controls (Table 1).

### 3.2. Genotype and Allele Distributions of *LXR-α* rs7120118 and *ABCA1* rs2230806 Polymorphisms

In terms of the rs7120118 and rs2230806 polymorphisms genotype and allelic frequencies in patients with DKD and controls, both groups were within Hardy-Weinberg equilibrium (*P* > .05), and their minor allele frequencies were over 5% (Table 2). Significant differences in the genotype and allele frequencies of the rs7120118 polymorphisms were detected between the DKD and DM groups (genotype, *P* = .027; allele, *P* < .001). In particular, the significant association between the minor allele (T) of rs7120118 and a decreased risk of DKD was identified in the allele (*P* < .001). The genotype frequencies of the rs2230806 were different between the two groups. No significant differences in allele frequencies of the rs2230806 were observed between the DKD and DM groups (genotype, *P* = .046; allele, *P* = .259).

### 3.3. Association of *LXR-α* rs7120118 and *ABCA1* rs2230806 Polymorphisms with DKD

Three kinds of statistical models were applied to test the genotypic associations of *LXR-α* rs7120118 and *ABCA1* rs2230806 polymorphisms with DKD (Table 3). We hypothesized that the minor alleles of both SNPs were the risk factors as compared with the common alleles. Multivariate logistic regression analysis was performed (Table 3). When the rs7120118 CC genotype was used as the reference, a significantly decreased risk of DKD was associated with the TC+TT genotype (TC+TT versus CC: odds ratio (OR), 0.684; 95% CI 0.501-0.933; *P* = .017) in the dominant model. When the rs7120118 CC homozygote genotype was used as the reference, the TC and TT genotypes were associated with a decreased risk of DKD (TC versus CC: OR, 0.719; 95% CI 0.522-0.990; TT versus CC: OR, 0.460; 95% CI 0.224-0.946; trend *P* = .043) in the additive model. With the rs7120118 CC+TC genotype as reference, we found that the TT genotype was not associated with the risk of DKD (TT versus CC+TC: OR, 0.582; 95% CI 0.292-1.158; *P* = .123) in the recessive model. After adjusting for sex and age, the results were similar. Only the rs2230806 AA+AG genotype was significantly associated with increased risk of DKD with the Holm-Bonferroni correction (*P* < .03). There was also no statistically significant association between *ABCA1* rs2230806 and the risk of DKD under the other genetic models.

### 3.4. Combined Effect of *LXR-α* rs7120118 and *ABCA1* rs2230806 Polymorphisms on DKD

The patients with the rs7120118 CC+rs2230806 AA genotype were used as reference. We found through multivariate logistic regression analysis that patients with rs7120118 CC+rs2230806 GG genotype showed an increased risk of DKD (OR, 2.531; 95% CI 1.262-5.078; *P* = .009; Table 4). No combined effect of other genotypes demonstrated an association with the risk of DKD. Results were similar after adjusting for age and sex (Table 4).

### 3.5. Genotype and Allele Distributions of *ABCA1* rs2230806 Polymorphisms in Patients without Hypercholesterolemia

We summarize the rs2230806 polymorphisms genotype and allelic frequencies in patients with or without hypercholesterolemia. The genotype frequencies of rs2230806 were different between DKD and DM without hypercholesterolemia groups; however, no significant differences in rs2230806 allele frequencies were observed in the two groups (genotype, *P* = .042; allele, *P* = .220). No other differences were demonstrated in the genotype and allele frequencies of the rs2230806 polymorphisms between the DKD and DM patients with hypercholesterolemia (Table 5).

Three statistical models were used to test the genotypic associations of *ABCA1* rs2230806 polymorphisms with DKD with or without hypercholesterolemia (Table 5). For rs2230806, the minor allele is the risk allele. With the rs2230806 CC+TC genotype as reference, the TT genotype was not associated with the risk of DKD (TT versus CC+TC: OR, 0.497; 95% CI 0 .233-1.058; *P* = .070) in the recessive model. Adjusting for age and sex did not change the results. Only the TC+TT genotype was associated with a significantly increased risk of DKD without hypercholesterolemia (*P* < .02).

Multivariate logistic regression analysis revealed that when the rs2230806 AA+AG genotype was used as the reference, a significantly increased risk of DKD was associated with the GG genotype (AA+AG versus GG: odds ratio (OR), 1.568; 95% CI 1.070-2.296; *P* = .021) in the recessive model. With the rs2230806 AA genotype was used as the reference, the AG+GG genotype was not associated with the risk of DKD (AG+GG versus AA: OR, 0.966; 95% CI 0.666-1.401; *P* = .854) in the dominant model. Adjusting for age and sex did not change the results. Only the GG genotype showed association with a significantly higher risk of DKD without hypercholesterolemia (*P* < .03).

## 4. Discussion

The major novel finding of the present study is that *LXR-α* rs7120118 is significantly associated with DKD in Han Chinese patients and confirms the association of its minor allele (C) with decreasing DKD risk. More importantly, we found that the patients with *LXR-α* rs7120118 CC and *ABCA1* rs2230806 GG genotype showed an increased risk of DKD.

Dyslipidemia has been identified to promote the progression of DKD [24]. Our previous research [13] and an increasing number of studies [25, 26] have placed further importance on cholesterol accumulation in the DKD kidney and are considered a risk factor of lipid metabolism disorder, which contributes to renal injury. As an important regulator of lipid metabolism, LXRs are the key mediator of lipid homeostasis, including maintaining lipid balance [27], and preventing or slowing atherosclerosis [28]. They are also important in regulating immunity [29] and exerting anti-inflammatory properties [30]. In our previous study, we found renal lipid deposition and kidney injury was aggravated by downregulation of LXR-*α*. However, the effectiveness of LXR agonists is limited by serious side effects, such as liver steatosis, hyperlipidemia, and impairment of neutrophil functions [31]. Interestingly, Akiyama et al. found that functional genes at homologous loci identified using human lipid GWASs responded to an animal model with high-fat and high-cholesterol diet intervention [32]. They observed a significant association of the *LXR-α* rs7120118 with serum lipid levels. Grzegorzewska et al. found that patients carrying the allele T at rs7120118 were lower in the hemodialysis patients with atherogenic dyslipidemia than in those without atherogenic dyslipidemia [10]. Similarly, our study showed that genotypic and allelic frequencies of rs7120118 were different between DM and DKD patients. And, patients with DKD had lower frequencies of rs7120118 T allele than the controls. In particular, the genotype and allele frequencies of rs7120118 SNP were also associated with the risk of DKD for different genetic models. In DKD patients, the rs7120118 TC+TT genotype was associated with a low risk of DKD (TC+TT versus CC), and the rs7120118 TC and TT genotypes were associated with a decreased risk of DKD (TC versus CC; TT versus CC) compared with DM patients. Therefore, dyslipidemia might be involved in the risk of *LXR-α* rs7120118 on DKD. However, Wu et al. demonstrated no significant association between rs7120118 genotype and risk of coronary heart disease in the Chinese Han population they studied [11].

Since ABCA1 promotes solubilization of lipids and their release, increasing ABCA1 should mediate efflux of free cholesterol from cells which is the early step in reverse cholesterol transport [33]. The molecular defect in the *ABCA1* gene results in Tangier disease, which is characterized by HDL deficiency, proteinuria, and premature atherosclerosis [34]. Additionally, some studies report that the genetic variants of ABCA1 are significantly associated with an individual's risk of developing coronary artery disease [35, 36]. In patients with DKD, a decrease in ABCA1 leads to an increase in cholesterol accumulation in renal tissues [34]. Similar changes in renal tissues were reported in diabetic apolipoprotein E knockout (apoE^−/−^) and db/db mice [30, 34, 37]. Additionally, because of the anti-inflammatory properties of ABCA1, its dysfunction tends to cause chronic low-grade inflammation in patients [38, 39]. Therefore, improving function or upregulating ABCA1 expression is sufficient to extenuate diabetic kidney injury. Studies have demonstrated that the *ABCA1* rs2230806 polymorphism is significantly associated with patients with severe dyslipidemia [40], such as coronary heart disease [41] and obesity [42], as well as with DM [19]. Meta-analysis by Jung et al. found that *ABCA1* rs2230806 polymorphism was significantly associated with DM in Asians [19]. Unfortunately, our results indicated that rs2230806 was not independently associated with the analyzed genotypes between the DM and DKD in a Chinese Han population.

As metabolite-sensing receptors, ABCA1 may have a close correlation with serum lipid levels. As such, we conducted subgroup analysis on hypercholesterolemia to test the genotypic associations of rs2230806 polymorphisms with DKD. Of further interest, the genotype frequencies of the rs2230806 were different between DM and DKD without hypercholesterolemia groups. The rs2230806 AG and GG genotypes were associated with a higher risk of DKD (AG versus AA; GG versus AA) compared with DM patients without hypercholesterolemia.

In conclusion, the current study suggests that *LXR-α* rs7120118 is significantly associated with the risk of DKD and confirms the association of its minor allele (T) with decreasing DKD risk. *ABCA1* rs2230806 is significantly associated with the risk of DKD and confirms its minor allele G as a higher risk factor for DKD without hypercholesterolemia. The association of LXR-*α*-ABCA1 is highly interesting in DKD. This genetic tool could identify high-risk DM patients who need closer monitoring to prevent or slow progression to DKD. Further studies are required, however, to investigate the biological mechanisms underlying this relationship.

## Figures and Tables

**Table 1:** Demographics and clinical characteristics of DM patients with and without kidney diseases.

Variables	DM (*n* = 346)^a^	DKD (*n* = 300)^a^	*P*
Age, *y*	60.0 (53.0, 67.0)	62.5 (54.0, 71.0)	.003
Sex, male (%)	57.51 (199 (1)/346)	61.33 (184 (1)/300)	.336
BMI, kg/m^2^	25.32 (23.08, 27.68)	25.80 (23.81, 28.18)	.021
Duration of diabetes, y	13.0 (10.0, 18.0)	15.0 (9.0, 20.0)	.149
History of hypertension (%)	51.16 (177/346)	78.0 (234/300)	<.001
SBP (mm Hg)	126.0 (120.0, 140.0)	140.0 (126.25, 150.0)	<.001
DBP (mm Hg)	80.0 (70.0, 80.25)	80.0 (74.0, 84.0)	.085
Scr (*μ*mol/L)	62 (52.0, 72.15)	84.55 (64.93, 141.93)	<.001
TC (mmol/L)	4.16 (3.47, 4.88)	4.28 (3.50, 5.04)	.093
TG (mmol/L)	1.49 (1.03, 2.23)	1.70 (1.20, 2.58)	.011
LDL-C (mmol/L)	2.40 (1.93, 3.03)	2.42 (1.88, 2.96)	.898
HDL-C (mmol/L)	1.01 (0.83, 1.21)	0.96 (0.78, 1.17)	.427

*P* < .05 indicates statistical significance. ^a^Data are shown as median (interquantile range) or %. Abbreviations: BMI: body mass index; DBP: diastolic blood pressure; HDL-C: high-density lipoprotein cholesterol; LDL-C: low-density lipoprotein cholesterol; SBP: systolic blood pressure; TC: total cholesterol; TG: triglyceride.

**Table 2:** Genotype and allele frequency of SNPs rs7120118 and rs2230806 between DM controls (*n* = 346) and DKD patients (*n* = 300).

	Genotype frequencies, *n* (%)				Allele frequencies, *n* (%)		
rs7120118	CC	CT	TT	*P*	C	T	*P*
DM	161 (46.5%)	160 (46.2%)	25 (7.2%)	.027^∗^	482 (69.7%)	210 (30.3%)	.011^∗^
DKD	168 (56.0%)	120 (40.0%)	12 (4.0%)		456 (76.0%)	144 (24.0%)	
rs2230806	AA	AG	GG	*P*	A	G	*P*
DM	86 (24.9%)	190 (54.9%)	70 (20.2%)	.046^∗^	362 (52.3%)	330 (47.7%)	.259
DKD	78 (26.0%)	139 (46.3%)	83 (27.7%)		295 (49.2%)	305 (50.8%)	

^∗^ *P* < .05. Abbreviations: DM: diabetes mellitus; DKD: diabetic kidney disease.

**Table 3:** Genetic model analyses of the association between the SNPs and DKD with adjustment for age and gender.

	Genetic models	Genotype	DM	DKD	Without adjustment		With adjustment^¶^	
					OR (95% CI)	*P*	OR (95% CI)	*P*
rs7120118	Additive	CC	161 (46.5%)	168 (56.0%)	1^#^		1^#^	
		TC	160 (46.2%)	120 (40.0%)	0.719 (0.522-0.990)	.043^∗^	0.711 (0.514-0.984)	.040^∗^
		TT	25 (7.2%)	12 (4.0%)				
	Dominant	CC	161 (46.5%)	168 (56.0%)	1^#^	.017^∗^	1^#^	.015^∗^
		TC+TT	185 (53.5%)	132 (44.0%)	0.684 (0.501-0.933)		0.677 (0.494-0.928)	
	Recessive	CC+TC	321 (92.7%)	288 (96.0%)	1^#^	.123	1^#^	.130
		TT	25 (7.2%)	12 (4.0%)	0.582 (0.292-1.158)		0.585 (0.293-1.171)	

rs2230806	Additive	AA	86 (24.9%)	78 (26.0%)	1^#^		1^#^	
		AG	190 (54.9%)	139 (46.3%)	0.807 (0.554-1.175)	.263	0.819 (0.560-1.197)	.302
		GG	70 (20.2%)	83 (27.7%)				
	Dominant	AA	86 (24.9%)	78 (26.0%)	1^#^	.739	1^#^	.842
		AG+ GG	260 (75.1%)	222 (74%)	0.941 (0.660-1.343)		0.964 (0.674-1.380)	
	Recessive	AA + AG	276 (79.8%)	217 (72.3%)	1^#^	.027^∗^	1^#^	.018^∗^
		GG	70 (20.2%)	83 (27.7%)	1.508 (1.048-2.171)		1.563 (1.081-2.260)	

^∗^ *P* < .05. ^¶^Adjustment for age and gender. ^#^Reference category (odds ratio, 1.0). Abbreviations: CI: confidence interval; DKD: diabetic kidney disease; DM: diabetes mellitus; ORs: odds ratios.

**Table 4:** The combined effect of *LXR-α* rs7120118 and *ABCA1* rs2230806 polymorphisms on DKD.

Genotypes		DM	DKD	Without adjustment		With adjustment^¶^	
rs7120118	rs2230806	346	300	OR (95% CI)	*P*	OR (95% CI)	*P*
CC	AA	46	45	1^#^	—	1^#^	—
CC	AG	97	81	0.854 (0.515-1.461)	.540	0.882 (0.529-1.469)	.629
CC	GG	18	42	2.385 (1.198-4.747)	.013^∗^	2.531 (1.261-5.078)	.009^∗^
CT+TT	AA	40	33	0.843 (0.455-1.564)	.589	0.853 (0.456-1.595)	.619
CT+TT	AG	93	58	0.638 (0.377-1.079)	.093	0.641 (0.377-1.092)	.102
CT+TT	GG	52	41	0.806 (0.451-1.440)	.466	0.845 (0.470-1.520)	.574

^∗^ *P* < .05. ^¶^Adjustment for age and gender. ^#^Reference category (odds ratio, 1.0). Abbreviations: DM: diabetes mellitus; DKD: diabetic kidney disease.

**Table 5:** Genetic model analyses of the association between SNP rs2230806 and DKD patients without hypercholesterolemia with adjustment for age and gender.

	Genetic models	Genotype	DM	DKD	*P*	Without adjustment		With adjustment^¶^	
						OR (95% CI)	*P*	OR (95% CI)	*P*
rs2230806	Additive	AA	82 (25.0%)	68 (25.7%)	.042^∗^	1^#^		1^#^	
		AG	181 (55.2%)	123 (46.4%)		0.819 (0.552-1.216)	.043^∗^	0.834 (0.560-1.243)	.031^∗^
		GG	65 (19.8%)	74 (27.9%)					
	Dominant	AA	82 (25.0%)	69 (25.7%)	—	1^#^	.854	1^#^	.972
		AG+GG	248 (75.0%)	196 (74.3%)		0.966 (0.666-1.401)		0.993 (0.682-1.447)	
	Recessive	AA+AG	276 (70.2%)	217 (72.1%)	—	1^#^	.021^∗^	1^#^	.013^∗^
		GG	65 (19.8%)	74 (27.9%)		1.568 (1.070-2.296)		1.634 (1.110-2.407)	

^∗^ *P* < .05. ^¶^Adjustment for age and gender. ^#^Reference category (odds ratio, 1.0). Abbreviations: CI: confidence interval; DKD: diabetic kidney disease; DM: diabetes mellitus; ORs: odds ratios.

## Data Availability

The data used to support the findings of this study are available from the corresponding authors upon request.

## References

[1] Saran R., Robinson B., Abbott K. C. (2017). US renal data system 2016 annual data report: epidemiology of kidney disease in the United States. *Americal Journal of Kidney Diseases*.

[2] Ogurtsova K., da Rocha Fernandes J. D., Huang Y. (2017). IDF diabetes atlas: global estimates for the prevalence of diabetes for 2015 and 2040. *Diabetes Research and Clinical Practice*.

[3] Gomes I. B., Porto M. L., Santos M. C. (2015). The protective effects of oral low-dose quercetin on diabetic nephropathy in hypercholesterolemic mice. *Frontiers in Physiology*.

[4] Hailing Z., Liang M., Meihua Y., Ping L. (2018). Association between MYH9 and APOL1 gene polymorphisms and the risk of diabetic kidney disease in patients with type 2 diabetes in a Chinese Han population. *Journal of Diabetes Research*.

[5] Ma L., Jiang Y., Kong X. (2019). Interaction of MTHFR C677T polymorphism with smoking in susceptibility to diabetic nephropathy in Chinese men with type 2 diabetes. *Journal of Human Genetics*.

[6] Opazo-Ríos L., Mas S., Marín-Royo G. (2020). Lipotoxicity and diabetic nephropathy: novel mechanistic insights and therapeutic opportunities. *International Journal of Molecular Sciences*.

[7] Wigger L., Casals-Casas C., Baruchet M. (2019). System analysis of cross-talk between nuclear receptors reveals an opposite regulation of the cell cycle by LXR and FXR in human HepaRG liver cells. *PLoS One*.

[8] Sundaram V. K., Massaad C., Grenier J. (2019). Liver X receptors and their implications in the physiology and pathology of the peripheral nervous system.. *International Journal of Molecular Sciences*.

[9] Nakagawa Y., Shimano H. (2018). CREBH regulates systemic glucose and lipid metabolism. *International Journal of Molecular Sciences*.

[10] Grzegorzewska A. E., Niepolski L., Świderska M. K. (2018). ENHO, RXRA, and LXRA polymorphisms and dyslipidaemia, related comorbidities and survival in haemodialysis patients. *BMC Medical Genetics*.

[11] Wu D. F., Yin R. X., Cao X. L. (2016). MADD-FOLH1 polymorphisms and their haplotypes with serum lipid levels and the risk of coronary heart disease and ischemic stroke in a Chinese Han population. *Nutrients*.

[12] Mouzat K., Molinari N., Kantar J. (2018). Liver X receptor genes variants modulate ALS phenotype. *Molecular Neurobiology*.

[13] Liu P., Peng L., Zhang H. (2018). Tangshen formula attenuates diabetic nephropathy by promoting ABCA1-mediated renal cholesterol efflux in Db/Db mice. *Frontiers in Physiology*.

[14] Suematsu Y., Kawachi E., Idemoto Y. (2019). Anti-atherosclerotic effects of an improved apolipoprotein A-I mimetic peptide. *International Journal of Cardiology*.

[15] Pedigo C. E., Ducasa G. M., Leclercq F. (2016). Local TNF causes NFATc1-dependent cholesterol-mediated podocyte injury. *Journal of Clinical Investigation*.

[16] Ganda A., Yvan-Charvet L., Zhang Y. (2017). Plasma metabolite profiles, cellular cholesterol efflux, and non-traditional cardiovascular risk in patients with CKD. *Journal of Molecular and Cellular Cardiology*.

[17] Teixeira M. D., Tureck L. V., do Nascimento G. A., de Souza R. L. R., Furtado-Alle L. (2020). Is it possible ABC transporters genetic variants influence the outcomes of a weight-loss diet in obese women?. *Genetics And Molecular Biology*.

[18] Du W., Hu Z., Wang L. (2020). ABCA1 variants Rs1800977 (C69T) and Rs9282541 (R230C) are associated with susceptibility to type 2 diabetes. *Public Health Genomics*.

[19] Jung D., Cao S., Liu M., Park S. (2018). A meta-analysis of the associations between the ATP-binding cassette transporter ABCA1 R219K (Rs2230806) polymorphism and the risk of type 2 diabetes in Asians. *Hormone and Metabolic Research*.

[20] Wang F., Ji Y., Chen X. (2019). ABCA1 variants Rs2230806 (R219K), Rs4149313 (M8831I), and Rs9282541 (R230C) are associated with susceptibility to coronary heart disease. *Journal of Clinical Laboratory Analysis*.

[21] World Health Organization (1999). *Definition, diagnosis and classification of diabetes mellitus and its complications: report of a WHO consultation. Part 1, Diagnosis and classification of diabetes mellitus*.

[22] National Kidney Foundation (2015). KDOQI clinical practice guideline for hemodialysis adequacy: 2015 update. *American Journal of Kidney Diseases*.

[23] Ma L., Yan M., Kong X. (2018). Association of EPHX2 R287Q polymorphism with diabetic nephropathy in Chinese type 2 diabetic patients. *Journal of Diabetes Research*.

[24] Yang L., Chu T. K., Lian J. (2018). Risk factors of chronic kidney diseases in Chinese adults with type 2 diabetes. *Scientific Reports*.

[25] Huang Y. C., Chen S. Y., Liu S. P. (2019). Cholesteryl ester transfer protein genetic variants associated with risk for type 2 diabetes and diabetic kidney disease in Taiwanese population. *Genes*.

[26] Hu J., Yang Q., Chen Z., Liang W., Feng J., Ding G. (2019). Small GTPase Arf6 regulates diabetes-induced cholesterol accumulation in podocytes. *Journal of Cellular Physiology*.

[27] Wang B., Tontonoz P. (2018). Liver X receptors in lipid signalling and membrane homeostasis. *Nature Reviews Endocrinology*.

[28] Ma C., Zhang W., Yang X. (2018). Functional interplay between liver X receptor and AMP-activated protein kinase *α* inhibits atherosclerosis in apolipoprotein E-deficient mice - a new anti-atherogenic strategy. *British Journal of Pharmacology*.

[29] Tavazoie M. F., Pollack I., Tanqueco R. (2018). LXR/ApoE activation restricts innate immune suppression in cancer. *Cell*.

[30] Smith T. K. T., Kahiel Z., LeBlond N. D. (2019). Characterization of redox-responsive LXR-activating nanoparticle formulations in primary mouse macrophages. *Molecules*.

[31] Souto F. O., Castanheira F., Trevelin S. C. (2020). Liver X receptor activation impairs neutrophil functions and aggravates sepsis. *Journal of Infectious Diseases*.

[32] Akiyama K., Liang Y. Q., Isono M., Kato N. (2015). Investigation of functional genes at homologous loci identified based on genome-wide association studies of blood lipids via high-fat diet intervention in rats using an in vivo approach. *Journal of Atherosclerosis and Thrombosis*.

[33] Xu Y., Xu Y., Zhu Y. (2020). Macrophage miR-34a is a key regulator of cholesterol efflux and atherosclerosis. *Molecular Therapy*.

[34] Ducasa G. M., Mitrofanova A., Mallela S. K. (2019). ATP-binding cassette A1 deficiency causes cardiolipin-driven mitochondrial dysfunction in podocytes. *Journal of Clinical Investigation*.

[35] Chen S. J., Tsui P. F., Chuang Y. P. (2019). Carvedilol ameliorates experimental atherosclerosis by regulating cholesterol efflux and exosome functions. *International Journal of Molecular Sciences*.

[36] Stock E. O., Ferrara C. T., O'Connor P. M. (2018). Levels of prebeta-1 high-density lipoprotein are elevated in 3 phenotypes of dyslipidemia. *Journal of Clinical Lipidology*.

[37] Yin Q.-h., Zhang R., Li L. (2016). Exendin-4 ameliorates lipotoxicity-induced glomerular endothelial cell injury by improving ABC transporter A1-mediated cholesterol efflux in diabetic apoE knockout mice. *Journal of Biological Chemistry*.

[38] Tan L., Liu L., Jiang Z., Hao X. (2019). Inhibition of microRNA-17-5p reduces the inflammation and lipid accumulation, and up-regulates ATP-binding cassette transporterA1 in atherosclerosis. *Journal of Pharmacological Sciences*.

[39] Asztalos B. F., Horvath K. V., Schaefer E. J. (2018). High-density lipoprotein particles, cell-cholesterol efflux, and coronary heart disease risk. *Arteriosclerosis, Thrombosis, and Vascular Biology*.

[40] Lu Z., Luo Z., Jia A. (2018). Associations of the ABCA1 gene polymorphisms with plasma lipid levels: a meta-analysis. *Medicine*.

[41] Fan Q., Zhu Y., Zhao F. (2020). Association of Rs2230806 in ABCA1 with coronary artery disease: an updated meta-analysis based on 43 research studies. *Medicine*.

[42] Yao M.-h., Guo H., He J. (2016). Interactions of six SNPs in ABCA1gene and obesity in low HDL-C disease in Kazakh of China. *International Journal of Environmental Research and Public Health*.

